# C57BL/6J and B6129F1 Embryo Transfer: Unilateral and Bilateral Transfer, Embryo Number and Recipient Female Background Control for the Optimization of Embryo Survival and Litter Size

**DOI:** 10.3390/ani10081424

**Published:** 2020-08-14

**Authors:** Sofia Lamas, Filipa Franquinho, Marlene Morgado, João R. Mesquita, Fátima Gärtner, Irina Amorim

**Affiliations:** 1 Animal Facility, i3S/ IBMC, Rua Alfredo Allen 208, 4200-135 Porto, Portugal; filipaff@ibmc.up.pt (F.F.); marlene.morgado@ibmc.up.pt (M.M.); fgartner@ipatimup.pt (F.G.); 2 Instituto de Biologia Molecular e Celular—IBMC, Rua Alfredo Allen 208, 4200-135 Porto, Portugal; 3 Epidemiology Research Unit (EPIUnit), Institute of Public Health, University of Porto, 4050-313 Porto, Portugal; jmesquita@outlook.com; 4 Institute of Biomedical Science Abel Salazar—ICBAS, R. Jorge de Viterbo Ferreira 228, University of Porto, 4050-313 Porto, Portugal; iamorim@ipatimup.pt; 5 Glycobiology in Cancer, IPATIMUP, R. Júlio Amaral de Carvalho, 45, 4200-135 Porto, Portugal

**Keywords:** embryo transfer, litter size, mouse unilateral and bilateral surgical transfers

## Abstract

**Simple Summary:**

Embryo transfer is a common procedure in rodent facilities related to rederivation protocols, recovery of cryopreserved embryos and production of genetically engineered animals. This procedure consists of the transfer of mouse embryos into the oviduct of a pseudopregnant recipient female in order to obtain live pups. The aim of this study is to further characterize the optimal conditions to perform embryo transfer using wild type strains and particularly the bilateral transfer. C57BL/6J and B6129F1 embryos were freshly collected and transferred to recipient females, after overnight culture to a 2-cell stage and tested for different conditions (unilateral and bilateral surgical procedures, variable number of embryos and reciprocity between recipient mother and embryo’s genetic background). The results achieved show that C57BL/6J transfers with a low number of embryos provide higher success rates when using unilateral transfers, but for bilateral transfers a minimum number of embryos seems to be necessary. B6129F1 presented similar results, but bilateral transfers were more effective with low number of embryos. These results allow a better planning of the embryo transfer procedure, considering low number of embryos and the choice of unilateral transfers as the ideal condition for an optimal outcome. This optimization has a positive impact on the 3R’s application: it can help to reduce the number of recipient and donor females and to improve recipient female’s welfare through the use of a less invasive technique.

**Abstract:**

Embryo transfer (ET) is a common procedure in rodent facilities. Optimizing this technique may help to reduce the number of animals, but little information is available regarding wild type strains and the conditions that affect embryo transfer. To explore this theme, 2-cell C57BL/6J embryos were transferred after overnight culture of freshly collected zygotes using different conditions: unilateral transfers using a total of 6, 8, 12, 15, 20 and 25 embryos were performed initially; then, this strain was also used for bilateral transfers using a total of 6, 12 and 20 embryos equally divided by the two oviducts. Groups of 25 embryos were not tested for the bilateral technique, since this condition produced the lower success rate when using the unilateral technique and 20 embryos would still represent a large number of embryos. A group of 2-cell B6129F1 embryos was also transferred using unilateral and bilateral ET with 6, 12 and 20 embryos. Crl:CD1(ICR) were used as recipient females for non-reciprocal transfers and C57BL/6J were used to test reciprocal transfers (only tested for six C57BL/6J unilateral transfers). Unilateral transfers using C57BL/6J mice produced higher success rates using six embryos, compared to the other groups transferred unilaterally (*p*-values between 0.0001 and 0.0267), but the mean number of pups per litter was not different among groups. Bilateral transfer produced higher number of pups when 20 embryos were divided by the two oviducts compared to six (*p* = 0.0012) or 12 (*p* = 0.0148) embryos, but with no differences in success rates. No statistical differences were found between the groups of B6129F1, but better results were obtained on bilateral transfers using a total of six embryos. For the strain tested (C57BL/6J), the uterine environment (Crl:CD1(ICR) or C57BL/6J recipient) does not impact the outcome of the technique. These results complement previous work published using genetically engineered mice strains and show that unilateral transfers using low number of embryos (6), produce better outcomes when compared to bilateral or unilateral transfers using more embryos. It also highlights differences between the outcome of bilateral transfers in the two strains tested. A set of historical data of genetically engineered mice at a C57BL/6J background was also included, confirming that lower embryo numbers are related to higher success rates. Together, the outcome of these experiments can be important to reduce the number of recipient and donor females, optimize embryo transfers and improve animal welfare discouraging the use of a more invasive technique.

## 1. Introduction

Embryo transfer (ET) is a key procedure related to assisted reproductive technologies (ART) in mice. The success of other techniques such as cryopreservation or in vitro fertilization (IVF), the efficiency of rederivation protocols and the production of genetically engineered mice all rely on the ability to produce a successful pregnancy after embryo transfer. Recently, several articles were published providing important insights about the factors that affect the ET efficiency, especially regarding the number of transferred embryos [1,2] and the surgical technique (bilateral or unilateral transfer) [3]. A reduced number of embryos transferred into the oviduct (between 8–12 embryos) [1] seems to produce a higher success rate measured as the ratio between the number of embryos transferred and the number of pups born. The work developed by Johnson et al. [4], using B6SJLF1 embryos, also concluded that transfers of 15 or more embryos did not result in more living fetuses.

Despite the variety of information available regarding ET, most of it is based on historical data from genetically engineered mice and it only considers a limited number of conditions: (1) data from genetically engineered mice is related to higher variability according to the different genetic modifications of each strain; (2) results obtained from specific number of embryos is lacking as the number of embryos analyzed is grouped in different intervals; (3) unilateral and bilateral transfers were only compared with each other when using the same number of embryos (15 to 18) and a wider group of conditions between unilateral and bilateral were not assessed; (4) bilateral transfers using small number of embryos were not evaluated; (5) genetic background reciprocity between the embryo and the receptive mother was also not explored. These gaps led us to test wild type strains in a larger group of experiments, testing a more complete and specific set of conditions whose results can be used as a basis for wild type strains ET. Although rederivation of mice is more commonly related to the transfer of genetically engineered mice between facilities or to produce a specific sanitary status within a facility, wild type strains are also frequently used for ET and little information is available about this subject in these strains.

Many factors can affect ET such as the pseudopregnancy state, the embryo quality and the genetic modifications, the background of the embryos or those related with the recipient female. Other factors such as the surgical technique (unilateral or bilateral) and the anesthetic and analgesic regimen selected are also relevant. Regarding pseudopregnancy, female receptivity to the male is higher at proestrus and visual observation of the vaginal mucosa [5] or synchronization of females through progesterone injection [6] are strategies to reduce the number of females needed to obtain pseudopregnant females. Embryo implantation after ET was described to be more efficient in females at diestrus when blastocyst transfer is performed [7]. Uterine receptivity and implantation rates are also strongly dependent on the embryo and endometrium interaction [8,9], since blastocysts regulate key molecules for the implantation process. Another factor for a successful ET is the embryo quality, which can be compromised either by in vitro maturation [10] or by superovulation. Superovulation, commonly used as a first step to obtain embryos for ET, has been described as having an evident impact on embryo development [11,12,13,14,15] and can affect the final ET result. The embryo genetic background [2,16,17] and genetic modifications [18] may also impact the implantation rate and the number of pups born. Finally, the surgical technique should also be considered when performing ET. The surgical technique selected may present variations regarding the anatomic location of the transfer (depending on the embryo developmental phase), although most commonly involving the oviduct. At this location, a variable number of embryos can be transferred unilaterally or bilaterally. The later demands a more invasive surgical approach, but with potential to produce higher number of living fetuses when compared to unilateral transfers [3]. Pregnancy rates are described as being higher when small number of embryos (<21) are used [2]. Furthermore, the ability to produce a pregnancy after ET has been reported as possible to be achieved with less than five embryos [2], 8 to 12 embryos [1] or less than 15 embryos [4] through unilateral transfer. Additionally, the analgesic and anesthetic protocol must be considered as a potential source of variation although several options are described as having no significant effect on the ET outcome [19,20,21,22,23], such as carprofen, used for this experimental work.

The aim of this experiment was to compare a different number of embryos from two defined mouse genetic backgrounds (C57BL/6J and B6129F1), transferred unilaterally and bilaterally and also to compare the most efficient condition using reciprocal and non-reciprocal embryo transfer methods. We started by analyzing all ET from genetically engineered mice at a C57BL/6J background performed in our facility during the last 2.5 years. Then, unilateral transfers in C57BL/6J using 6, 8, 12, 15, 20 and 25 embryos were tested. The experiment was then repeated using bilateral transfers with a total of 6, 12 and 20 embryos, equally divided by both oviducts. Unilateral and bilateral ET was then repeated by transferring 6, 12 and 20 embryos using a different wild type strain donor (B6129F1). The main goal was to understand the impact of both the embryo’s number and the surgical technique used for ET in wild type strains and to complement the data available using genetically engineered mice historical data, especially regarding bilateral transfers using small number of embryos. These results may contribute both to improve animal welfare and reduce the number of females involved in this process. It will also allow a practical optimization of ET protocols in rodent animal facilities.

## 2. Materials and Methods

The experimental work was approved by the i3S Animal Welfare and Ethics Review Body (AWERB) and submitted for approval at the Portuguese Competent Authority (DGAV), (project reference 2017_03). Experiments were performed ensuring animal welfare and according to the European legislation (Directive 63/2010).

### 2.1. Animals

Animals were born and kept at the i3S animal facility and were maintained in regular conditions: temperature was maintained between 20 °C and 24 °C and humidity between 45% and 65%; distillated water was provided ad libitum as well as autoclaved feed (2014S Envigo diets); a corn cob bedding was used and environmental enrichment (paper tube roles) and paper for nesting were provided inside the cages. The Crl:CD1(ICR), C57BL/6J and 129/SvPasCrl colonies were originally bought to Charles River; breeders are replaced frequently in order to maintain the genetic integrity of the strains. Females were housed in 1264C Eurostandard Type II (groups of up to 5 females) or 1290D Eurostandard Type III (groups of 6 to 10 females) cages; males were housed in 1264C Eurostandard Type II cages (1 animal per cage for the breeding). C57BL/6J and 129/SvPasCrl were free from the following agents: MHV, EDIM, MPV, MVM, PVM, Sendai virus, TMEV, Ectromelia, LCMV, MAD-1 and 2, Mouse *Cytomegalovirus*, Reovirus, *Bordetella bronquiseptica*, *Citrobacter rodentium*, *Clostridium piliforme*, *Corynebacterium kutsheri*, *Cryptosporidium* spp., *Mycoplasma pulmonis*, *Pasteurella* spp., *Pseudomonas aeruginosa*, *Salmonella* spp., *Helicobacter* spp., *Staphylococcus aureus*, *Streptococcus pneumoniae*, *Streptobacillus moniliformis*, *Klebsiella pneumoniae*, ectoparasites (*Myobia, Radfordia* and *Myocoptes* spp.), *Eimeria* spp., *Entamoeba muris*, *Giardia* spp., *Spironucleus muris*, *Tritrichomonas muris*, *Aspiculuris tetraptera* and *Syphacea obvelata* and *muris*. Occasional positives were found at quarantine (origin of animals from the genetically engineered mice at a C57BL/6J background) for *Pasteurella* spp., *Helicobacter* spp., *Klebsiella pneumoniae* and *Staphylococcus aureus* as animal origin differs among the two experiments.

### 2.2. Experimental Groups

**Figure 1:**
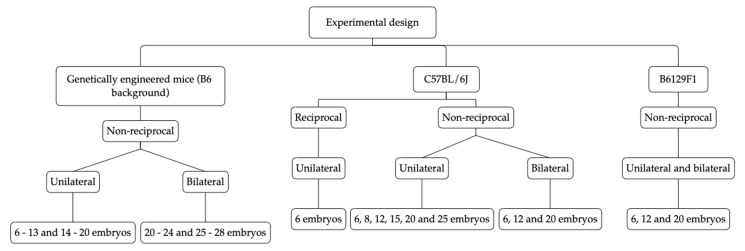
Experimental design and groups.

This work includes 3 sets of experiments (summarized in Figure 1) one set involving historical data of genetically engineered mice at a C57BL/6J background, using unilateral and bilateral non-reciprocal embryo transfers; a second set of prospective experiments involving reciprocal and non-reciprocal unilateral and bilateral transfers with C57BL/6J wild-type mice; a third group of prospective experiments involving non-reciprocal unilateral and bilateral transfers with B6129F1 wild-type mice. The choice for the number of embryos tested in the bilateral condition was based on the results of the unilateral transfer.

Genetically engineered mice at a C57BL/6J background historical data: a total of 39 sessions of ET performed unilaterally and 35 sessions of bilateral ET from a total of 36 genetically engineered mice strains at a C57BL/6J background were analyzed, using non-reciprocal transfers. The number of 2-cell embryos transferred for the unilateral technique was between 6 and 22 and for the bilateral transfer was between 15 and 28. For easier comparison, unilateral transfers were grouped in 6–13 and 14–22 transfers of 2-cell embryos. Bilateral transfers were grouped in transfers using 20–24 and 25–28 embryos.

Data from C57BL/6J mice: a controlled experiment using wild-type mice (C57BL/6J) was done. For this experiment embryo transfers were performed according to the following conditions: non-reciprocal embryo transfer was performed using C57BL/6J 2-cell embryos transferred in groups of 6, 8, 12, 15, 20 and 25 unilaterally or 6, 12 and 20 embryos transferred bilaterally into CD1 pseudopregnant females (equally by the two oviducts); reciprocal embryo transfers were performed using 6 C57BL/6J embryos, transferred unilaterally to C57BL/6J pseudopregnant females. Transfers were performed during the morning and each female was allocated to a group in a random way.

Data from B6129F1 mice: another set of experiments was performed using B6129F1 embryos. Unilateral and bilateral non-reciprocal transfers using 6, 12 and 20 embryos were performed and CD1 pseudopregnant females were used as recipients. Transfers were also performed during the morning and each female was allocated to a group in a random way.

### 2.3. Superovulation

PMSG (ProSpec, HOR 272) and hCG (ProSpec, HOR 250) were diluted at a dose of 50 IU in sterile water and frozen at −20 °C until further use. Before injection, aliquots were thawed and diluted in sterile water to get a 100 µL dosage of 5 IU for intraperitoneal injection.

PMSG and hCG were given at an interval of 48 h. Hormones were given at 3:00 p.m. at a light cycle of 12:12-h light:dark cycle, lights on at 7:00 a.m. At the time of hCG injection, females were mated with males overnight and checked for plug in the following morning.

For the genetically modified mice at a C57BL/6J background historical data, males used were between 4 and 6 months of age, with proved fertility and donor females between 3 and 5 weeks of age.

For the C57BL/6J experiment, males were between 4 and 6 months of age, with proved fertility; males were used once per week allowing a one week resting period between matings. C57BL/6J donor females were between 3 and 5 weeks of age.

The B6129F1 experiment used 129/SvPasCrl males between 3 and 5 months of age and a similar breeding scheme as the one described for the C57BL/6J males. These males were crossed with C57BL/6J donor females between 3 and 5 weeks of age.

### 2.4. Embryo Collection

To collect embryos at 0.5 dpc, plug positive females were euthanized in the morning after the mating by cervical displacement. Oviducts were collected in M2 media [24] and, after tearing the ampulla, the cumulus cells surrounding the zygotes were digested in M2 supplemented with 0.5-mg/mL hyaluronidase (Sigma-Aldrich, H4272) for less than 1 min. After digestion, embryos were washed in 50 µL M2 drops, followed by several washes in 50 µL KSOM drops (EmbryoMax^®^ KSOM Mouse Embryo Media, ref. MR-020P-5 F) and incubated in the same media drops using the same volume covered with mineral oil (Mineral oil, Sigma-Aldrich, ref. M8410). In the following morning, embryos at 1.5 dpc were washed in M2 and transferred to a pseudopregnant Crl:CD1(ICR) female or C57BL/6J (in case of testing reciprocity).

### 2.5. Embryo Transfer

Genetically engineered mice at a C57BL/6J background historical data: Crl:CD1(ICR) females were used as recipient females for the embryos (non-reciprocal). Before mating, CD1 female mice were housed in 1290D Eurostandard Type III cages in groups of 8 to 10 females. For the embryo transfer, 0.5 dpc pseudo-pregnant females (age between 5 to 10 weeks) were obtained by crossing surgically vasectomized CD1 males with demonstrated infertility (8 to 16 weeks of age) with CD1 females. Surgical anesthesia was obtained with a mixture of intraperitoneal ketamine (80 mg/kg, Clorketam) and medetomidine (1 mg/kg, Sededorm); carprofen (0.5 mg/kg, Rymadil) was administered subcutaneously before the surgery and kept during the first 48 h after surgery. A heating pad, eye ointment (Siccafluid, Unidoses 2.5 mg/g, Laboratoires THÉA) and aseptic technique were applied before surgery. The surgical procedure was started when the withdrawal reflex was not present. Atipamezole was used for female’s recovery (1 mg/kg, Revertor). For this group, only unilateral transfers were performed, always on the left oviduct. Briefly, a 0.5 to 1 cm incision on the left dorsal side of the abdominal wall followed by an incision on the peritoneal wall were performed in order to expose the ovary fat. Using blunt forceps, the ovarian fat was removed from the peritoneal cavity to expose the oviduct and ovary. Bulldog forceps were then applied to the ovarian fat and the oviduct was identified under the stereomicroscope. Only females with a large ampullae were used for the embryo transfer. Once the ampullae was identified, embryos were placed inside a pulled glass capillary with minimum M2 media, followed by a small air bubble. A small cut using spring scissors was performed in the area before the ampullae, after the infundibulum, just large enough to introduce the capillary. After capillary insertion, the embryos were placed inside the oviduct and the ovary and oviduct were returned to the abdominal cavity. The peritoneum and the skin were sutured using polyglycolic acid, 6-0 suture line.

Data from C57BL/6J and B6129F1 wild-type mice: For these experiments’ recipient females (Crl:CD1(ICR) for the non-reciprocal and C57BL/6J for the reciprocal groups) were anesthetized using isoflurane (5% for induction and 2%–3% for maintenance with oxygen at 1L/min for induction and 0.2 to 0.3 L/min for maintenance). Carprofen was also used for analgesia and the same surgical procedure described above for unilateral transfers of genetically engineered mice was used. For the unilateral transfers using C57BL/6J as recipient females (reciprocal), the anesthesia, analgesia and surgical procedure were the same as described for unilateral transfers of wild type C57BL/6J and B6129F1 mice. C57BL/6J recipient females were used between 10 and 16 weeks of age and mated with surgically vasectomized CD1 (8 to 16 weeks) males with demonstrated infertility to induce pseudopregnancy.

Bilateral transfers were performed using the same anesthetic and analgesic protocol described for the genetically engineered mice. The surgical procedure consisted in doing an incision over the lumbar area (0.5 to 1 cm) and exposition of the peritoneal wall on both sides. A second incision was performed at the peritoneum wall from the left side and a similar procedure to the described for the unilateral transfer was done for the embryo transfer. The procedure was repeated on the right side and, after closing both peritoneum incisions, the skin was sutured using polyglycolic acid, 6-0 line. Bilateral transfers were all performed using non-reciprocal transfers with a total of 6, 12 and 20 embryos in C57BL/6J and B6129F1, equally divided by the two oviducts.

### 2.6. Statistical Analysis

GraphPad Prism version 8.00 for Mac, GraphPad Software, La Jolla, CA, USA, www.graphpad.com was used for statistical analysis. We assessed data for normality using the D’Agostino–Pearson normality test whenever the number of observations allowed its application; alternatively, the Kolmogorov–Smirnov test was used when the sample did not allow the use of the first test. We used a Mann–Whitney test to compare the historical data. In this group only the number of pups born was compared as two independent groups considering unilateral and bilateral transfers, as the variation between the mean number of embryos used for unilateral and bilateral transfer was high and success rates and the number of pups could not be compared. Success rates were only compared using the same test for the two unilateral groups and a second independent test was performed for the two bilateral groups. For the C57BL/6J experiment, a one-way ANOVA followed by a Tukey’s multiple comparison test was done between the unilateral transfers and a second set of comparisons was used for the bilateral group. Reciprocal and non-reciprocal transfers using 6 embryos were equally analyzed using a Student’s *t*-test. Transfers using the same number of embryos transferred bilaterally and unilaterally were subsequently analyzed using a two-way ANOVA, as well as the B6129F1 transfers. The comparison between the C57BL/6J and B6129F1 transfers was performed using a three-way ANOVA. A *p*-value of 0.05 or less was considered as a statistically significative result.

## 3. Results

Genetically engineered mice at a C57BL/6J background historical data: we aimed to compare ET using different number of embryos for unilateral and bilateral transfers using historical data from genetically modified mice. Since the mean number of embryos transferred was distinct between unilateral and bilateral methods, results were grouped according to the number of embryos used. For unilateral and bilateral transfers, two groups were established as stated in Table 1. We observed that the mean number of pups born after unilateral transfers using 6 to 13 embryos was lower comparing to unilateral transfers using 14 to 22 embryos (*p* = 0.0196). No statistical differences were found between the two groups for bilateral transfers (*p* = 0.3292). Success rates, defined as the percentage of embryos that gives origin to a live pup, between the two unilateral transfer groups and between the bilateral transfer groups were not significantly different (*p* = 0.1865 and *p* = 0.0724, respectively). Pregnancy rates were also not significantly different between the 4 groups (*p* = 0.159).

**Figure 2:**
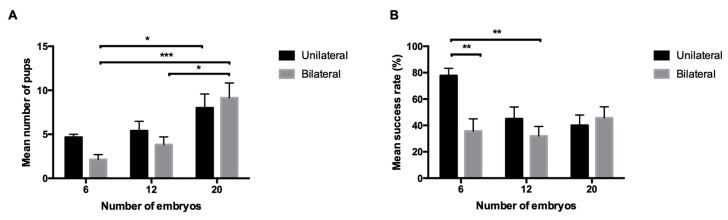
(**A**) Mean number of pups after unilateral and bilateral transfers using 6, 12 and 20 C57BL/6J embryos. Statistically significant differences were found between 6 embryos unilateral group and 6 and 12 embryos bilateral; (**B**) success rates for unilateral and bilateral transfers using 6, 12 and 20 embryos. Statistically significant differences were found between 6 embryos bilateral and 20 embryos unilateral and bilateral and between 12 and 20 embryos bilateral. Values are mean ± SEM. * *p* < 0.05, ** *p* < 0.01, *** *p* < 0.001.

Data from C57BL/6J: We aim to further understand the impact of both the embryo’s number and the surgical technique used for ET in a wild type strain (C57BL6/J), especially regarding bilateral transfers using small number of embryos. Mean number of pups born and the success rates are shown in Table 2. As for non-reciprocal unilateral transfers, the mean number of pups born was not statistically different between the different unilateral groups, meaning that the size of the litter does not increase significantly with an increase in the number of embryos transferred (*p*-values between 0.1079 and 0.999). On the other hand, when comparing the success rate of unilateral transfers, statistically significant differences were found between transfers of 6 and 15 embryos (77.8% vs. 42.7%, *p* = 0.0062), between 6 and 20 (77.8% vs. 40%, *p* = 0.0029), between 6 and 25 (77.8% vs. 24%, *p* < 0.0001), between 6 and 12 (77.8% vs. 45%, *p* = 0.0117) and between 8 and 25 embryos (55% vs. 24%, *p* = 0.0267). For the non-reciprocal bilateral transfers significant statistical differences on the mean number of pups born were found between 6 and 20 embryos (*p* = 0.0012) and between 12 and 20 embryos (*p* = 0.0148); the mean number of pups was not statistically different between 6 and 12 embryos (*p* = 0.5821). Regarding the success rate, no significant differences were found between any of the non-reciprocal bilateral groups (*p*-values between 0.5083 and 0.9486). Unilateral and bilateral non-reciprocal embryo transfers using the same number of embryos were also compared: a statistically significant increase in the success rate was found when comparing 6 embryos unilateral transfer (77.8%) to: 6 embryos bilateral transfer (35.7%, *p* = 0.0065), 12 embryos bilateral transfer (31.9%, *p* = 0.0038), 20 embryos bilateral transfer (45.7%, *p* = 0.0038) and 20 embryos unilateral (40%, *p* = 0.0405). The number of pups was significantly different between 6 embryos bilateral group and 20 embryos unilateral (*p* = 0.0166) and bilateral (*p* = 0.0008) groups and between 12 and 20 embryos bilateral groups (*p* = 0.0257). Then, we tested reciprocity to assess whether the genetic background of recipient females was associated with an increased success rate. No statistical differences were detected between 6 C57BL/6J embryos transferred into a CD1 or C57BL/6J pseudopregnant female (*p* = 0.1828). Figure 2 represents the results for the C57BL/6J mice using 6, 12 and 20 embryos unilateral and bilateral transfers. The mean number of embryos needed to obtain one live pup are represented in Table 3.

**Figure 3:**
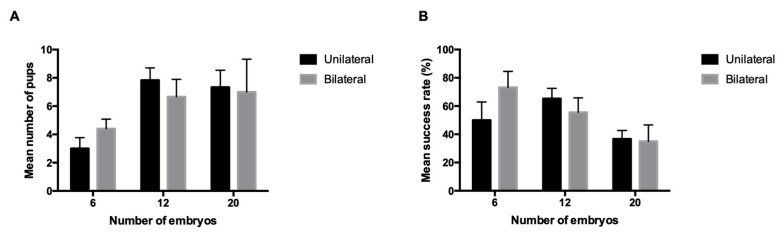
(**A**) Mean number of pups after unilateral and bilateral transfer for B6129F1 transfer groups (6, 12 and 20 embryos); (**B**) success rates for unilateral and bilateral transfer for B6129F1 transfer groups (6, 12 and 20 embryos). Values are mean ± SEM. No statistically significant differences were found between the groups.

Data from B6129F1: We aim at evaluating the mean number of pups and the success rates in a second hybrid strain. Only non-reciprocal transfers were performed for this strain and no statistical differences were found between the success rates (*p*-values between 0.2317 and 0.9999) or between the mean number of pups (*p*-values between 0.1231 and 0.9999) (Table 4, Figure 3). The mean number of embryos needed to obtain one live pup are represented in Table 3.

Comparison between C57BL/6J and B6129F1 results: we aim at comparing the unilateral and bilateral ET techniques using 6, 12 and 20 embryos in the two different strains. The comparison of these conditions between C57BL/6J and B6129F1 strains, revealed no significant differences between the two strains for the success rates, but significant differences were found for the number of pups in transfers using 6 C57BL/6J embryos bilateral and 12 B6129F1 embryos unilateral (*p* = 0.0325) and between 6 B6129F1 embryos unilateral and 20 C57BL/6J bilateral (*p* = 0.0144).

## 4. Discussion

This study presented the differences in the outcome of ET using two wild type strains, with a particular interest in bilateral transfers, that were until now only characterized for 15 to 18 embryos and using genetically engineered mouse strains [2]. This work is also the first to characterize ET in wild type strains as, to date, all the available data were focused on historical data obtained from genetically engineered mice. A set of historical data corresponding to genetically engineered mice at a C57BL/6J background was also included. These data are not directly comparable to the prospective study using C57BL/6J and B6129F1, but can, in part, be compared to the already available data on this topic. Our results show, for the genetically engineered mice at a C57BL/6J background, that lower number of pups are obtained when using unilateral non-reciprocal transfers with 6 to 13 2-cell embryos compared 14 to 22 embryos (*p* = 0.0196), but the success rates are not significantly different. This result is in accordance with the findings of Gonzalez-Jara et al. [1], who concluded that smaller number of embryos transferred unilaterally are related to a higher success rate in genetically engineered mice. Despite both data correspond to C57BL/6J embryos, our results revealed a mean number of pups per litter higher than the previously mentioned author. Both studies used CD-1 as receptive females, but the anesthesia and analgesia were distinct (isoflurane versus ketamine and medetomidine); the embryo collection method was also different (our investigation involves incubation). Nevertheless, the simple fact that the animal’s genetic alterations were distinct may explain the small differences obtained. The anesthesia and analgesia regimen involved in both studies has been described as having no significant effects on embryo implantation [23,25]. However, superovulation and incubation may affect in vitro and in vivo embryo development [13], and the in vitro culture seems to have a lower impact when compared to a stimulated uterine environment after PMSG/hCG administration [14,26], which can, in part explain the higher mean number of pups herein obtained.

The prospective study using C57BL/6J revealed no differences between the groups, when comparing the mean number of pups in unilateral transfers. However, the success rates for the unilateral transfer using C57BL/6J were significantly different between those using 6 embryos unilaterally with all the other groups. These results corroborate previously published data showing that a low number of embryos has a better outcome than a higher number [1]. The embryo effort, measured as the number of embryos needed to obtain one live pup, is lower when using smaller number of embryos for the transfer: the unilateral transfer using 6 embryos needs a mean number of 1.3 embryos to produce one live pup. Notwithstanding, this number is substantially increased when using 12 (2.8), 15 (2.4) or 20 (3) embryos. Despite being able to produce a normal pregnancy rate, the 25 embryos group, resulted in the lowest success rate and the higher embryo effort to produce one live pup (4.5). Obtaining better results in conditions that involve a lower number of embryos may be related to the uterine capacity and ability to have free space for implantation, as described by Johnson et al. in 1996 [4]. This author used a different strain and only evaluated the number of implantation sites and reabsorptions, without a full pregnancy, but described 11–12 fetuses as the maximum uterine capacity. A slightly higher value (15) was found in our work (a litter of 15 pups obtained after a 20 unilateral transfer using C57BL/6J). The result for the group involving 15 to 25 embryos may also be related to the fact that mice have a duplex uterus that includes a divided cervix, that avoids embryo’s uterine migration [27]. This characteristic may be the cause for the lower success rates related to ET using high number of embryos, as the uterine capacity is exceeded. Overall, the mean number of pups obtained in the different groups is higher when compared to the work published using unilateral transfers from Gonzalez-Jara et al. and Dorsch et al. [1,2], except for transfers using 25 embryos (7.5 pups described by Gonzalez-Jara et al. versus 6 pups obtained in our prospective work). These differences related to higher mean number of pups, may be related to the fact that the prospective study presented here used wild type strains, with a lower variability and possibly higher embryo capacity to survive after ET. In addition, other results available in the literature grouped the embryos in different classes, impairing proper comparisons. The same can be said regarding the success rates, our work using C57BL/6J wild type animals is related to slightly higher pregnancy rates when compared to the work of Gonzalez-Jara et al. 

Bilateral transfers are most commonly used when a large number of embryos is intended to be transferred, but little information was available regarding lower number of embryos. The prospective study using C57BL/6J revealed significant differences between the mean number of pups using 6 and 12 embryos and between 12 and 20 embryos, with higher mean numbers of pups being related to the 20 embryos group transfer. For this strain, better success rates were also related to higher number of embryos, a contradictory result when considering the numbers obtained for unilateral transfers. Groups of 3 to 5 embryos were already described as enough to produce pregnancy [2] but this effect was, until now, only evaluated for unilateral transfers.

Using a different strain (B6129F1), a F1 hybrid, no relevant differences between the conditions tested for the unilateral transfer were detected, revealing again that lower embryo numbers are able to produce more efficient outcomes. Bilateral transfers with B6129F1 also did not produce significant differences, but, in contrast to the results obtained with C57BL/6J for bilateral transfers, B6129F1 displayed a higher success rate when lower number of embryos were used. This effect on bilateral transfers, although not statistically significant, may suggest that there are strain dependent differences that, perhaps with a larger set of animals could be better understood. The size of B6129F1 pups may explain the inability to have better outcomes when using a larger number of pups [28]. C57BL/6J, on the other hand, are smaller and the better results obtained with 20 embryos bilateral transfers may be related to this strain characteristic. It is interesting to notice that 6 embryos using the bilateral technique produced two times more pups in B6129F1, when compared to C57BL/6J. The genetic background may, again, explain the differences found as both the mother and embryos background may affect the embryo development [29]. Mouse embryo implantation demands a fine tune between the embryo and the mother uterine cavity, with the balance between estrogens and progesterone being essential for implantation to occur [30,31,32]. There is also evidence that embryo manipulation, such as embryo culture and the use of superovulation have detrimental effects on embryo survival [14,33,34,35,36,37], as already stated. These effects may have distinct levels when different strains are considered, such as different strains survive differently to other type of manipulations, such as microinjection [38].

Regarding the background of the recipient female, no differences were found when embryos were transferred into an oviduct of the same background (B6) or into a different background (CD1). Considering that CD1 females tend to have good maternal skills, it is more efficient to use a non-reciprocal transfer in this case. This may be questionable if other backgrounds or genetic modifications are considered It is known that the survival of embryos is more dependent on the mother background than on the embryo background [39] and other authors also described differences between C3H, NMRI and DBA mice as recipient females [16].

These results can significantly impact the number of embryo donor’s and maximize the collected embryo’s use. For the C57BL/6J, six embryos transferred unilaterally produced the most efficient success rate (77.8%) with expected mean numbers of 4.6 pups. B6129F1 had the best outcome when six embryos were transferred bilaterally (mean number of pups of 4.4 and a success rate of 73.3%). Although no significant differences were found, B6129F1 seems to have better results when 20 embryos are used bilaterally, when compared to the same condition in C57BL/6J. Recipient females used can equally be optimized by planning the number of needed transfers according to the expected size of litters described. Bilateral transfers do not seem to offer any advantage from a success rate point of view and require a more complex, time consuming and invasive option. Thus, the choice for a unilateral transfer improves animal welfare. In the particular case of the B6129F1 strain, the bilateral transfer when having low number of embryos (six embryos) seems to offer some advantages although these differences were not significative.

## 5. Conclusions

In conclusion, for wild type C57BL/6J, better results after embryo transfer were obtained when using six embryos unilaterally: success rates of unilateral transfers decrease when the number of embryos increases. The mean number of pups does not increase proportionally to the number of embryos transferred when using unilateral transfers, but, in bilateral transfers, higher mean numbers of pups are obtained when 20 embryos are introduced. Thus, when using bilateral transfers in C57BL/6J, more than 12 embryos should be considered as a total for the transfer. B6129F1 behave similarly, with better outcomes when using lower number of embryos unilaterally. Nevertheless, bilateral transfers in this strain had better results with a lower number of embryos, perhaps due to the size of B6129F1 pups. Bilateral transfer does not seem to offer any obvious advantage and represents a more invasive technique with a probable higher negative impact on animal welfare. To date, no information was available regarding ET of wild type strains, which may serve as a more reliable basis for ET planning. An effort to reduce the number of animals used must be done by optimizing common techniques such as ET. Together with other information related to the best practices when using superovulation and incubation, these results can help the entire process optimization and significantly reduce the number of donor females needed for ET, helping to improve the efficiency of ET by increasing the number of live pups obtained after transfer.

## Figures and Tables

**Table 1:** Genetically engineered mice at a C57BL/6J background transfers, grouped by number of embryos.

Reciprocity	Surgical Technique	Number of Transfers	Total Number of Embryos Transferred	Pregnancy Rate (%)	Mean Number of Pups Born per Litter ^a^
Non-reciprocal	Unilateral	17	6 to 13	100	5.5 ± 0.4
		22	14 to 22	77.3	7.1 ± 0.4
	Bilateral	23	20 to 24	73.9	6.4 ± 0.4
		12	25 to 28	83.3	5.5 ± 0.6

Total number of transfers, number of embryos transferred, pregnancy rates and mean number of pups born after unilateral and bilateral transfers using genetically engineered mice at a C57BL/6J background. For bilateral transfers, embryos were divided equally by the two oviducts. ^a^ Values are mean ± SEM.

**Table 2:** C57BL/6J transfer groups and results.

Reciprocity	Surgical Technique	Total n of Embryos Transferred	Number of Transfers	Number of Pregnant Females	Pregnancy Rate (%)	Number of Pups Born per Litter ^a^	Success Rate (%) ^a^
Non-reciprocal	Unilateral	6	7	6	85.7	4.6 ± 0.3	77.8 ± 5.6
		8	7	5	71.4	4.4 ± 0.6	55 ± 7.5
		12	7	5	71.4	5.4 ± 1.08	45 ± 9
		15	7	5	71.4	6.4 ± 0.4	42.7 ± 2.7
		20	6	5	83.3	8 ± 1.6	40 ± 7.9
		25	6	5	83.3	6 ± 0.9	24 ± 3.6
	Bilateral	6	7	7	100	2.1 ± 0.6	35.7 ± 9.2
		12	6	6	100	3.8 ± 0.9	31.9 ± 7.3
		20	7	7	100	9.1 ± 1.7	45.7 ± 8.4
Reciprocal	Unilateral	6	7	6	85.7	3.8 ± 0.5	63.9 ± 8

Number of transfers, pregnant females, mean number of pups and success rate (number of pups born/number of embryos transferred) for C57BL/6J are summarized at table for each of the experimental conditions (reciprocal and non-reciprocal; unilateral and bilateral and according to the number of embryos transferred). ^a^ Values are mean ± SEM.

**Table 3:** Embryo effort for the generation of one live pup using C57BL/6J and B6129F1.

Reciprocity	Surgical Technique	Total Number of Embryo’s Transferred	C57BL/6J ^a^	B6129F1 ^a^
Non-reciprocal	Unilateral	6	1.3 ± 0.1	3.1 ± 0.9
		8	1.8 ± 0.5	–
		12	2.8 ± 0.8	1.6 ± 0.2
		15	2.4 ± 0.17	–
		20	3 ± 0.7	3.7 ± 1.3
		25	4.5 ± 0.6	–
	Bilateral	6	3.8 ± 0.8	1.6 ± 0.4
		12	4.7 ± 1.6	2.1 ± 0.3
		20	3.3 ± 1.2	4.6 ± 1.5
Reciprocal	Unilateral	6	1.7 ± 0.3	–

Embryo effort measured as the number of embryos needed to obtain one live pup using C57BL/6J and B6129F1. ^a^ Values are mean ± SEM.

**Table 4:** B6129F1 transfer groups and results.

Reciprocity	Surgical Technique	Total n of Embryos Transferred	Number of Transfers	Number of Pregnant Females	Pregnancy Rates (%)	Number of Pups Born per Litter ^a^	Success Rate (%) ^a^
Non-reciprocal	Unilateral	6	7	6	85.7	3 ± 0.8	50 ± 12.9
		12	7	6	85.7	7.8 ± 0.9	65.3 ± 7.2
		20	7	6	85.7	7 ± 2.2	36.7 ± 6
	Bilateral	6	7	5	71.4	4.4 ± 0.7	73.3 ± 11.3
		12	7	6	85.7	6.7 ± 1.2	55.6 ± 10.2
		20	6	5	83.3	7.3 ± 2.2	35 ± 11.6

Number of transfers, pregnant females, mean number of pups and success rate (number of pups born/number of embryos transferred) for B6129F1 strain are summarized at table for each of the experimental conditions (non-reciprocal; unilateral and bilateral and according to the number of embryos transferred). ^a^ Values are mean ± SEM.
